# Larger visual changes compress time: The inverted effect of asemantic visual features on interval time perception

**DOI:** 10.1371/journal.pone.0265591

**Published:** 2022-03-22

**Authors:** Sandra Malpica, Belen Masia, Laura Herman, Gordon Wetzstein, David M. Eagleman, Diego Gutierrez, Zoya Bylinskii, Qi Sun

**Affiliations:** 1 Universidad de Zaragoza, Zaragoza, Spain; 2 Adobe, Inc., Mountain View, CA, United States of America; 3 Department of Electrical Engineering, Stanford University, Stanford, CA, United States of America; 4 Department of Psychiatry, Stanford University School of Medicine, Stanford, CA, United States of America; 5 New York University, New York, NY, United States of America; Anglia Ruskin University, UNITED KINGDOM

## Abstract

Time perception is fluid and affected by manipulations to visual inputs. Previous literature shows that changes to low-level visual properties alter time judgments at the millisecond-level. At longer intervals, in the span of seconds and minutes, high-level cognitive effects (e.g., emotions, memories) elicited by visual inputs affect time perception, but these effects are confounded with semantic information in these inputs, and are therefore challenging to measure and control. In this work, we investigate the effect of asemantic visual properties (pure visual features devoid of emotional or semantic value) on interval time perception. Our experiments were conducted with binary and production tasks in both conventional and head-mounted displays, testing the effects of four different visual features (spatial luminance contrast, temporal frequency, field of view, and visual complexity). Our results reveal a consistent pattern: larger visual changes all shorten perceived time in intervals of up to 3min, remarkably contrary to their effect on millisecond-level perception. Our findings may help alter participants’ time perception, which can have broad real-world implications.

## Introduction

Our perception of time crucially affects our ability to process our surroundings, make predictions, and act in real and simulated environments. Manipulations of time perception have been leveraged in broad applications. For instance, patients undergoing medical procedures can experience shorter durations of time, helping them cope with anxiety [1]; urban planning is also affected by time perception, since perceived waiting times in transit can be altered by the presence of basic amenities, indirectly affecting productivity [2]; and reaction times have to be considered in the context of public safety, especially for those who are driving [3]. Time perception can also be used as a proxy to measure performance in professional training, particularly when considering fitness for duty [4]. Evidence suggests that varied biological mechanisms might be involved in the perception of different temporal durations [5], including sub-second range (millisecond timing), seconds-to-hours range (interval timing), and a 24-hour range (circadian timing).

In this work, we define *asemantic visual features* as lower-level factors that are intrinsically related to visual processing (e.g., luminance contrast, temporal frequency), but not high-level cognitive aspects like emotions. In contrast, we denote factors related to high-level cognitive aspects as *semantic features*. Millisecond range time perception has been reported as being affected by purely visual, low-level patterns [6], which could be disentangled from other high-level cognitive aspects [7]. For instance, a positive correlation was found between visual magnitude and time perception at the millisecond level: higher-magnitude stimuli (e.g., stronger luminance, larger sizes, etc.) were judged to last longer in a prospective paradigm (participants knew beforehand that they would be making a temporal judgement [8]). In comparison, interval timing is the most common duration in high-order, real-world tasks, and it is known to be altered by semantic features such as emotional valence, arousal, cognitive load, or zeitgebers [1, 9, 10]. Manipulating the visual content to be sad or funny [9], varying the task difficulty (e.g., asking participants to solve either a 2D or 3D puzzle) [1], or having participants notice the changes in the illumination of a scene due to the position of the sun [10] are all examples of manipulations to semantic features that have been reported to affect time perception at interval timing durations.

In these previous works, high cognitive load or arousal generally shortened perceived time at the level of multiple seconds to more than one hour. However, since features like emotional valence and arousal are partially subjective and individualized, it is difficult to consistently and universally affect time perception by manipulating semantic features. It is much more practical to manipulate asemantic visual features, since they can be explicitly controlled (e.g., by computer rendering photo-realistic stimuli). Despite this, the effects of asemantic visual features on interval timing remain significantly under-explored. This is likely because, within interval timing, it is difficult to entirely disentangle low-level from high-level features (e.g., manipulating color is known by content creators to elicit different emotions [11]). While past work has thus mainly considered the effects of high-level semantic features on time perception, we manipulate asemantic visual features by carefully curating the experimental stimuli.

### In this paper, we aim to answer the fundamental question “whether and how do *asemantic* visual features alter human time perception at the interval level?

Specifically, we investigate the prospective paradigm informing participants from the beginning that they will be making judgements related to time. This contrasts with the retrospective paradigm, in which participants report on the passage of time at the end of a task, without being informed in advance that a temporal judgement will be solicited. Contrary to retrospective judgements that rely on memory [12], we rely on prospective judgements to simulate ecologically valid applications and understand how time perception is affected in *real-world* scenarios in which people are actively aware of the passage of time.

Our experiments are designed to test the following hypothesis: differences in the *magnitude* of asemantic visual features should alter time perception in prospective judgements at interval timing durations. We initially deploy our experiments with two different display interfaces: conventional displays (CDs) and virtual reality head-mounted displays (HMDs). Since we find similar (and consistent with previous work) trends in time perception for both display conditions, we favor HMD setups for the subsequent experiments. HMDs offer full immersion, allowing for more realistic visual simulation with computer-generated stereo stimuli (thus depth cues), larger fields of view effects [13], and free viewing with dynamic head motion. They enable full control over the visual stimuli displayed to participants, regardless of whether they remain still or move. Thus, time perception can be studied in more natural conditions while maintaining control over the virtual environment [10, 14–16]. Besides, time perception is an important factor to consider in VR applications when designing a better human-computer interface. This has been emerging as a critical demand in the medical field, where, for example, the presentation of distracting content through HMDs has proven to shorten the perceived duration of chemotherapy treatments [1]. We believe that time perception manipulations could have a similar effect in training or simulation applications, allowing for longer exposure sessions.

The emotional valence, semantic, or other high-level cognitive aspects of the presented content cannot always be manipulated at will without affecting the experience or the goal of a given application. Instead, we propose the manipulation of asemantic visual features to alter time perception without affecting high-level cognitive aspects. In that regard, we study four visual features pertaining to three abstraction levels: low-level spatiotemporal visual properties (*spatial luminance contrast* and *temporal frequency*), mid-level display-related properties (*field of view*), and high-level cognitive aspects (*visual complexity*, understood as the number of independent visual sources in a scene).

In our prestudies, we replicated an experiment [6] that studied the effect of asemantic visual stimuli at the millisecond timing level as a baseline and found similar results across different viewing conditions (CD and HMD). However, directly extending the millisecond experimental design to longer intervals did not reveal consistent results. We term this phenomenon a *perceptual break*. This perceptual break may be due to the different neural mechanisms that have been found to be in charge of processing different temporal magnitudes in mammals [5, 17] where millisecond timing is processed ‘automatically’ [18] while interval timing is ‘cognitive’ (by engaging attention and working memory [19]). A perceptual break can be defined as the point where the perceptual process changes abruptly. In our case, we found that the perceived effect of visual changes on time perception was different depending on the duration of the tested period, particularly when comparing millisecond (less than one second) to interval (multiple seconds) timing.

**Fig 1:**
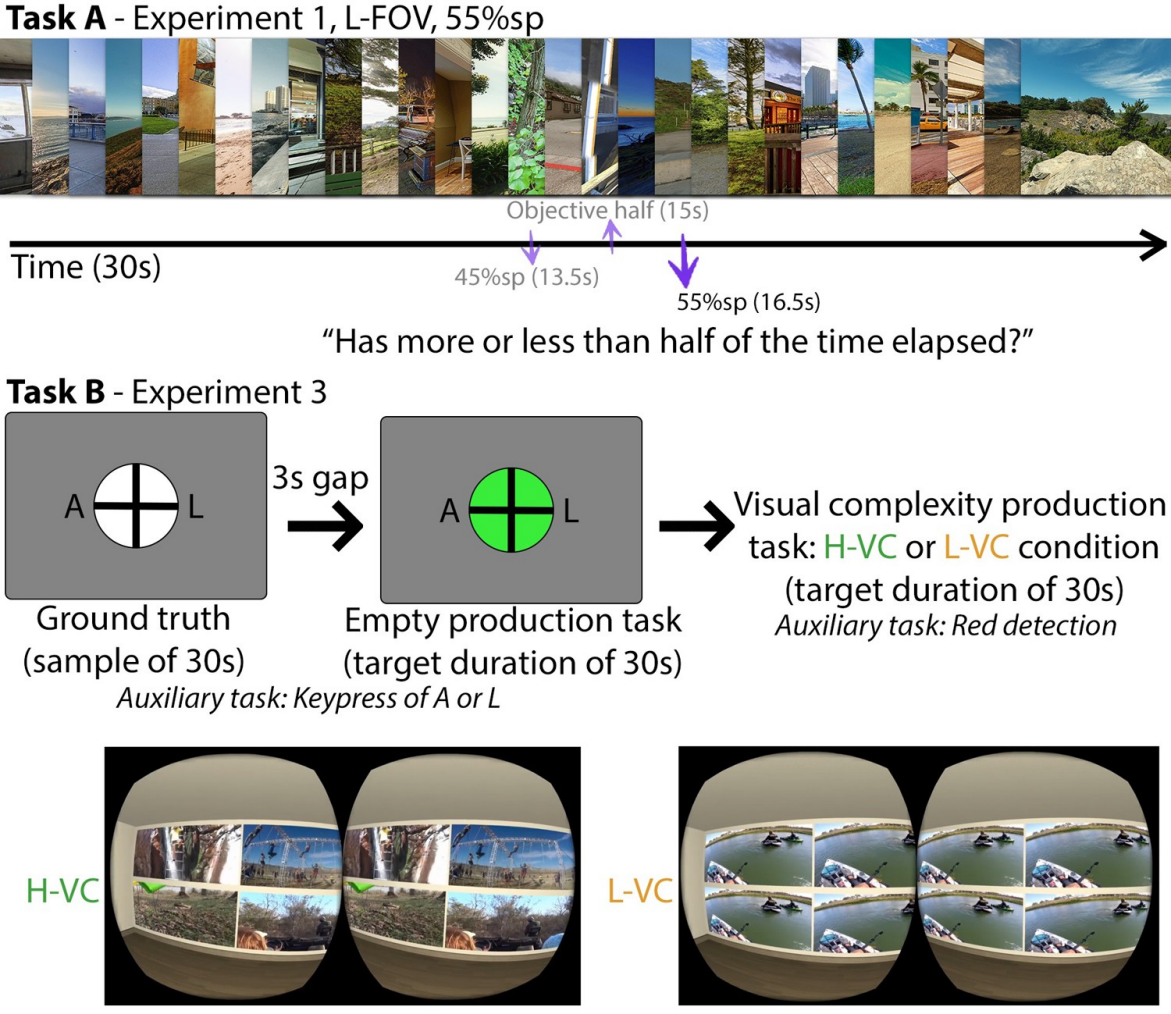
Illustration of the experiments flow. ***Task A***) Binary task used in *Experiments 1–2*. Illustrative example for a single trial (L-FOV condition in Experiment 1 at the 55% sp, see Section 2.1). During a trial, participants indicated whether “more than half” or “less than half” of the duration had passed, at a temporal sampling point that was 55% of the total duration (16.5s). ***Task B***) The double production task used in *Experiment 3*. First participants were presented with an empty scene for 30s (*ground truth*) while performing an auxiliary task (detecting the presence of A or L on screen with a corresponding keypress). Then they were asked to press a key after 30s had elapsed (*empty production task*). At this point, the empty scene was replaced with a scene in the H-VC or L-VC condition (high or low visual complexity, respectively). After the change of scene, participants had to press a key when 30 more seconds had elapsed (*visual complexity production task*). In the H-VC condition, the four screens of the virtual room displayed different videos at the same time. In the L-VC condition, the four screens presented the same video synchronously.

Following, we present our main experiment (Experiment 1) and additional experiments that suggest how the effect behaves under varied conditions. With *Experiment 1* we tested how differences in the *magnitude* of luminance contrast, temporal frequency, and field of view affect time perception using 30s intervals. In *Experiment 2* we tested intervals of up to five minutes to check whether the effects found in *Experiment 1* hold for longer intervals. Both *Experiments 1 and 2* make use of *half-duration judgements*, in which participants are interrupted part way through a trial and asked to estimate whether more or less than half of the full timing interval has already elapsed (binary response). Finally, we design *Experiment 3* with the aim of providing a quantitative estimate of the temporal distortion, which is based on a numerical estimation of the passage of time, in seconds. See Fig 1 for a visual summary of the experimental procedures.

Results across all of our experimental conditions reveal a common trend: duration of time is consistently perceived as shorter when high magnitude levels of each visual feature are present, regardless of viewing conditions or the experiment task. We found this trend to hold across intervals ranging from 30 seconds to three minutes. In particular, larger spatial luminance contrast, higher temporal frequency, larger FoV, and more complex visual content consistently shorten participants’ prospective time judgments when compared with lower levels of the same visual features.

## Materials and methods

### Ethics statement

All experiments were carried out following the Helsinki recommendations, and ensuring data anonymization. Our experimental protocols were approved by the Consejo de Gobierno (Government Council) of Universidad de Zaragoza. At the beginning of the experiments, participants gave informed consent and were made aware of the possibility of stopping or abandoning the experiments at any point at their will.

### Experimental design

The objectives of our experiments were to analyze the effects of asemantic visual features on prospective time judgements for interval timing (several seconds to minutes range). With that aim, we designed two different tasks (A and B) and carried out four experiments with different sets of participants for a total population size of 168 participants. In the following, we describe each of the experiments in detail. A video showing an accelerated version of the experimental procedure for Tasks A and B can be found in S1 Movie, while a complete visualization of our results can be found in Figs 3–6 and Tables 2 and 3.

### 1. Prestudies

**Fig 2:**
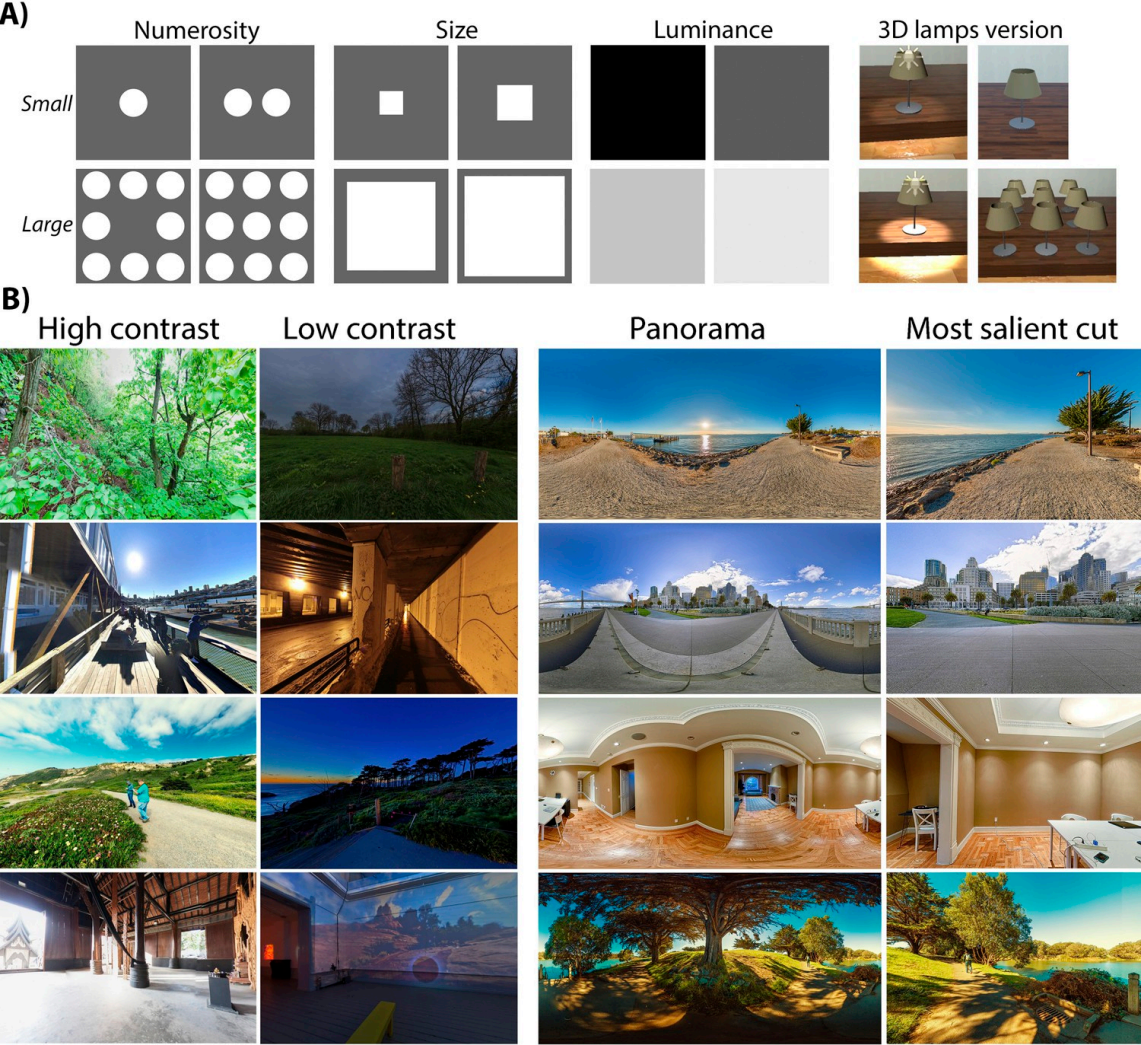
Stimuli samples. **A)** Stimuli used in the prestudies. *Left*: The 2D, grayscale stimuli we adapted from Xuan et al [6]. *Right*: The 3D lamps used in our follow-up attempts to directly extend the procedure of Xuan et al. to interval timing. **B)** Illustrative examples of the stimuli used in our experiments depicting natural scenes. From left to right, in each column: high contrast (H-CON), low contrast (L-CON), 360° panoramas (H-FOV), and their associated most salient crops (L-FOV). Fig 1 (Task B) also shows an example of high and low visual complexity (H-VC and L-VC).

Previous work reported that the emotional reactions (level of arousal and valence) experienced by observers while viewing short movie clips were driving factors in time perception, while the viewing conditions (display type) were not [9]. Following the work of van der Ham et al. [9], we ran an initial study to validate whether their findings held by replacing emotional factors with asemantic visual features. With that aim, we first replicated a previous experiment on millisecond timing that measured the effects of visual magnitude on time perception [6] with simple 2D stimuli of abstract patterns (Fig 2A), and compared the results obtained on a conventional display, or CD (first viewing condition) with those obtained on an HMD (second viewing condition). This study was completed by five participants who observed both viewing conditions. Participants judged the duration of pairs of “small” and “large” stimuli, presented for 600-937ms using a pairwise forced-choice comparison. The stimuli were simple and abstract. Following Xuan et al.’s notation, “small”/“large” indicates the level of the visual features in the stimuli. For example, a square with low luminance is referred to as “small”, while a square with high luminance is referred to as “large”. Size, numerosity, and digits were similarly categorized. As in Xuan et al.’s work [6], we denote as *incongruent* short durations with “large” stimuli, and correspondingly, long durations with “small” stimuli, since their temporal and visual magnitudes do not match. According to the results reported in the paper, participants were inclined to judge larger stimuli as longer, regardless of their actual duration: in CDs (first viewing condition) the mean difference in error rates was 20% on average between incongruent and congruent trials, with incongruent trials having higher error rates.

We then extended Xuan et al.’s work by using an HMD (second viewing condition) and observed the same trend. We found a mean difference in error rates of 37.6% between congruent and incongruent stimuli across the five participants, with higher error rates in incongruent trials, consistent with the results trend that was found in the original work using CDs. These findings are consistent with those of van der Ham et al. [9], indicating that similar results can be obtained using HMDs and CDs. This, together with previous work, is indicative that the trend of the effect of visual stimuli magnitude on time perception should hold between HMDs and CDs.

In the following, we tried to directly extend the same experimental design to interval timing. However, our follow-up attempts to extend the work of Xuan et al. [6] to longer interval durations (5s, 10s, and 30s) did not exhibit the same effect (the mean difference in error rates between congruent and incongruent stimuli dropped to 5.2%). This trend was consistent with the *perceptual break* between millisecond and interval duration magnitudes evidenced in previous works [5]. Debriefing sessions also revealed participants’ loss of engagement while observing simple stimuli for long periods, which might have contributed to the small differences observed in error rates between congruent and incongruent stimuli. We proceeded to design a more realistic scenario with 3D computer-generated models of real objects (specifically, lamps) instead of the original 2D stimuli of simple, abstract patterns. In this extended experiment, we again observed the same trends at millisecond intervals but did not find significant differences at longer durations (6.4% mean difference in error rate). However, participants suggested on post-study free written form questionnaires that they preferred the more realistic stimuli. This motivated us to design our main experiment (Experiment 1) and subsequent studies leveraging more complex and realistic stimuli (Fig 2B) to maintain participant engagement throughout the experiments and attention in the timing tasks.

### 2. Interval timing experiments

In the prestudies, we found that the effects of the absolute visual magnitude (i.e., “big” vs “small” stimuli in Xuan et al.’s experiments [6]) observed with millisecond timing did not seem to replicate when extended to interval durations, possibly due to the aforementioned perceptual break [5]. We thus design our interval timing experiments based on this knowledge, as well as inspired by Montague’s theory of time perception [20]: “*if [*…*] perceptual space and time magnitudes are essentially relative matters […]*, *the amount of objective time or change which appears to be present at any one moment will be measured by its ratio to the subjective change which accompanies it*”. In other words, it is not the absolute visual value that affects time perception, but rather the perceived changes across visual stimuli.

In our experiments, we study how changes in the *magnitude* of four different asemantic visual features affect time perception, ranging from lower to higher levels of abstraction: luminance contrast and temporal frequency (low-level), field of view (mid-level), and visual complexity (high-level). Our stimuli of choice are quickly-varying frames (in the case of both static images and short video sequences), the design choice motivated by our prestudy suggesting that a reasonable amount of variation was required to maintain user engagement, critical to obtaining a reliable measure of time perception. Further, in potential application scenarios, it is likely that, at the interval timing durations we are considering, variation in the visual input will naturally be present.

Extending the notions of “big” and “small” from Xuan et al.’s studies, in all our experiments we similarly have two *magnitude* levels: *high* and *low* (see Fig 2). Regardless of the viewing conditions (different display types or visual features being manipulated) throughout our experiments, the *high* level is designed to exhibit a larger absolute value of the visual feature than the *low* level. The changes elicited by the visual feature will also be generally perceived as larger in the high level than in the low level. We define visual changes as spatiotemporal per-pixel variations. In this sense, we assume that a sequence of stimuli with higher luminance contrast (larger per-pixel variations), temporal frequency (faster variations), field of view and visual complexity (both with more numerous variations) will all cause larger visual changes than their lower-level counterparts.

**Fig 3:**
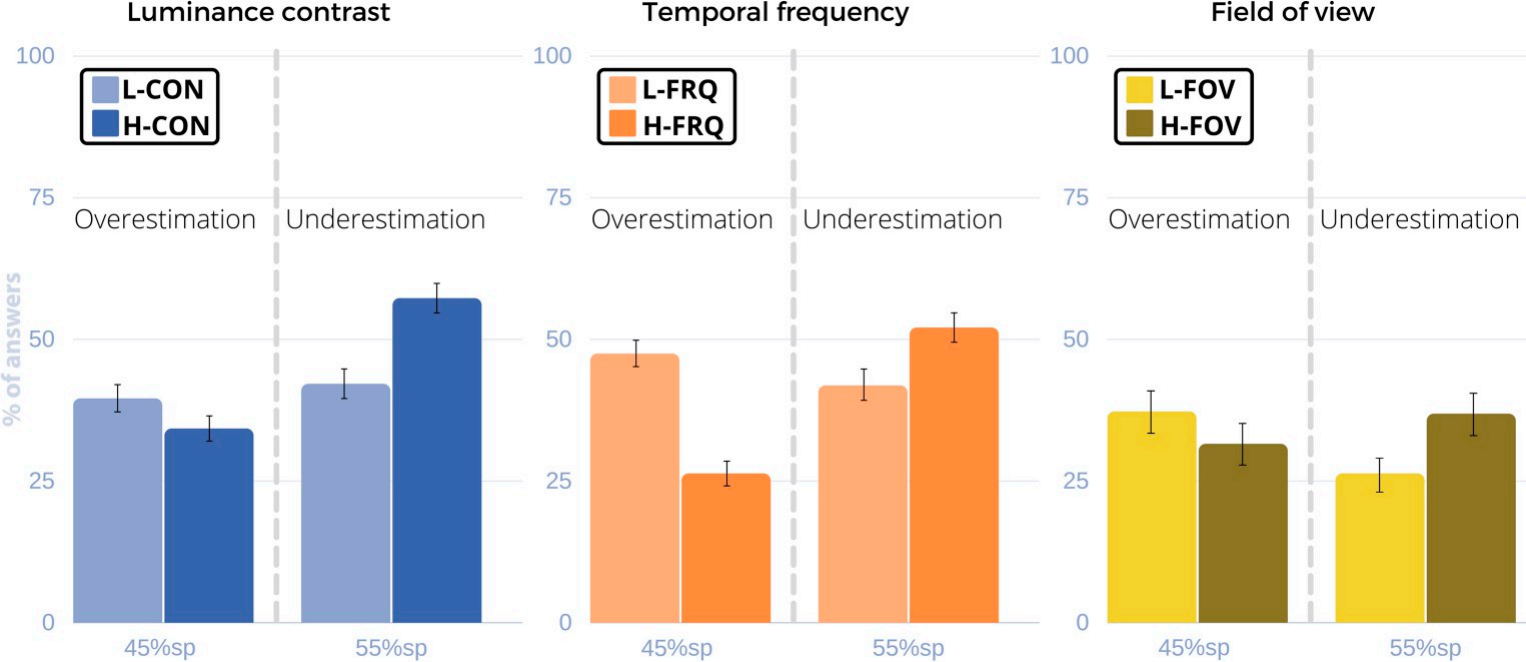
Results for Experiment 1. From left to right: Aggregation of trials by luminance contrast, temporal frequency and field of view conditions. Y axis: % of answers. X axis: 45% and 55% sampling points (sp). The displayed bars of each graph correspond to p_over,45_ at the 45% sp (overestimation) and to p_under,55_ at the 55% sp (underestimation). The percentage of correct answers for each case is complementary to the displayed value in the graph (p_corr,45_+p_over,45_ = 100% and p_corr,55_+p_under,55_ = 100%). Note that overestimation is always more frequent in low magnitude levels and underestimation in high magnitude levels, regardless of the visual feature.

**Fig 4:**
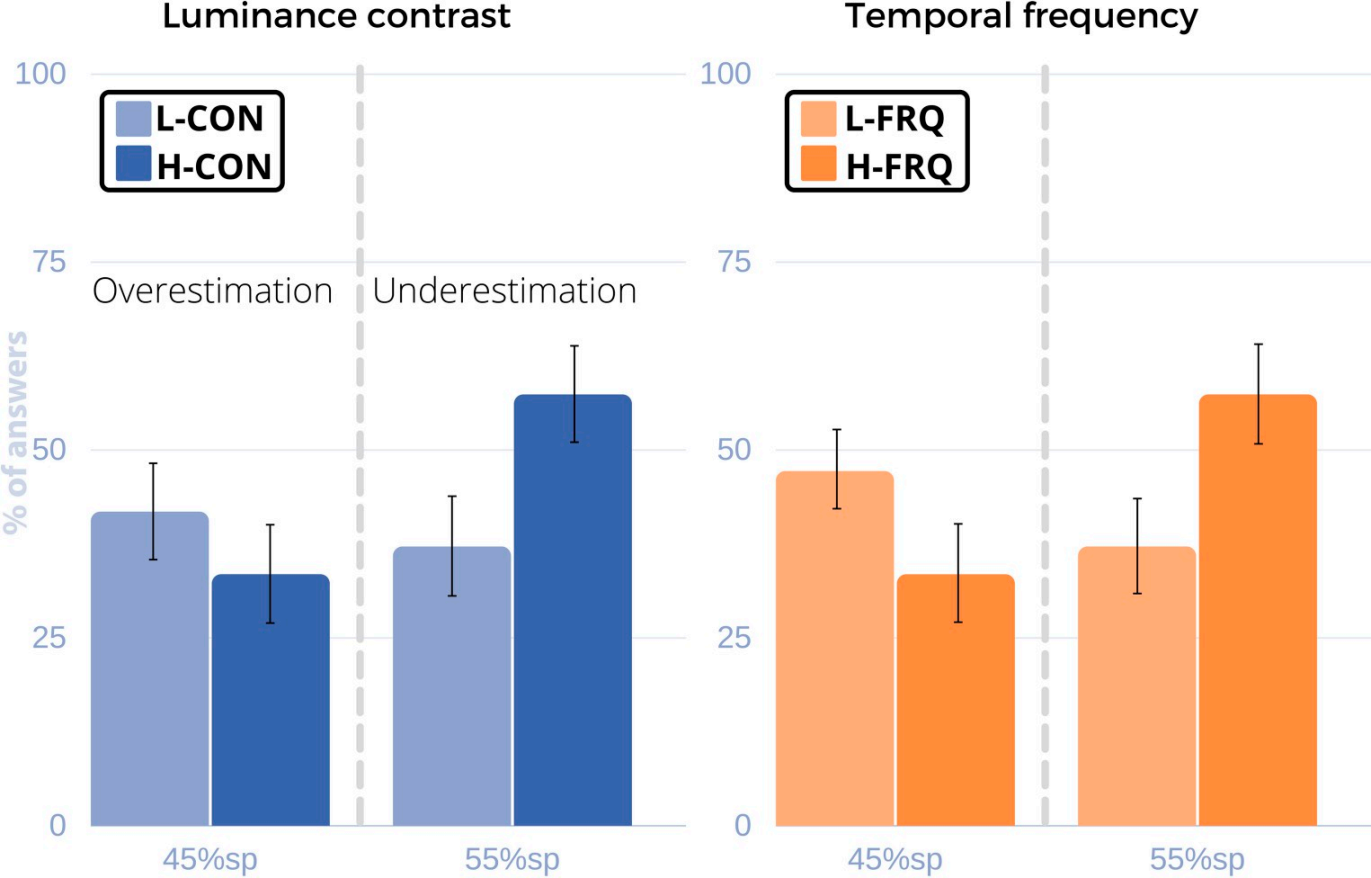
Results for the follow-up replication study with conventional displays. Left: Luminance contrast trials. Right: Temporal frequency trials. Y axis: % of answers. X axis: 45% and 55% sampling points (sp). The displayed bars of each graph correspond to p_over,45_ at the 45% sp (overestimation) and to p_under,55_ at the 55% sp (underestimation). The percentage of correct answers for each case is complementary to the displayed value in the graph (p_corr,45_+p_over,45_ = 100% and p_corr,55_+p_under,55_ = 100%). Like in Experiment 1, overestimation is always more frequent in low magnitude levels and underestimation in high magnitude levels, regardless of the visual feature.

**Fig 5:**
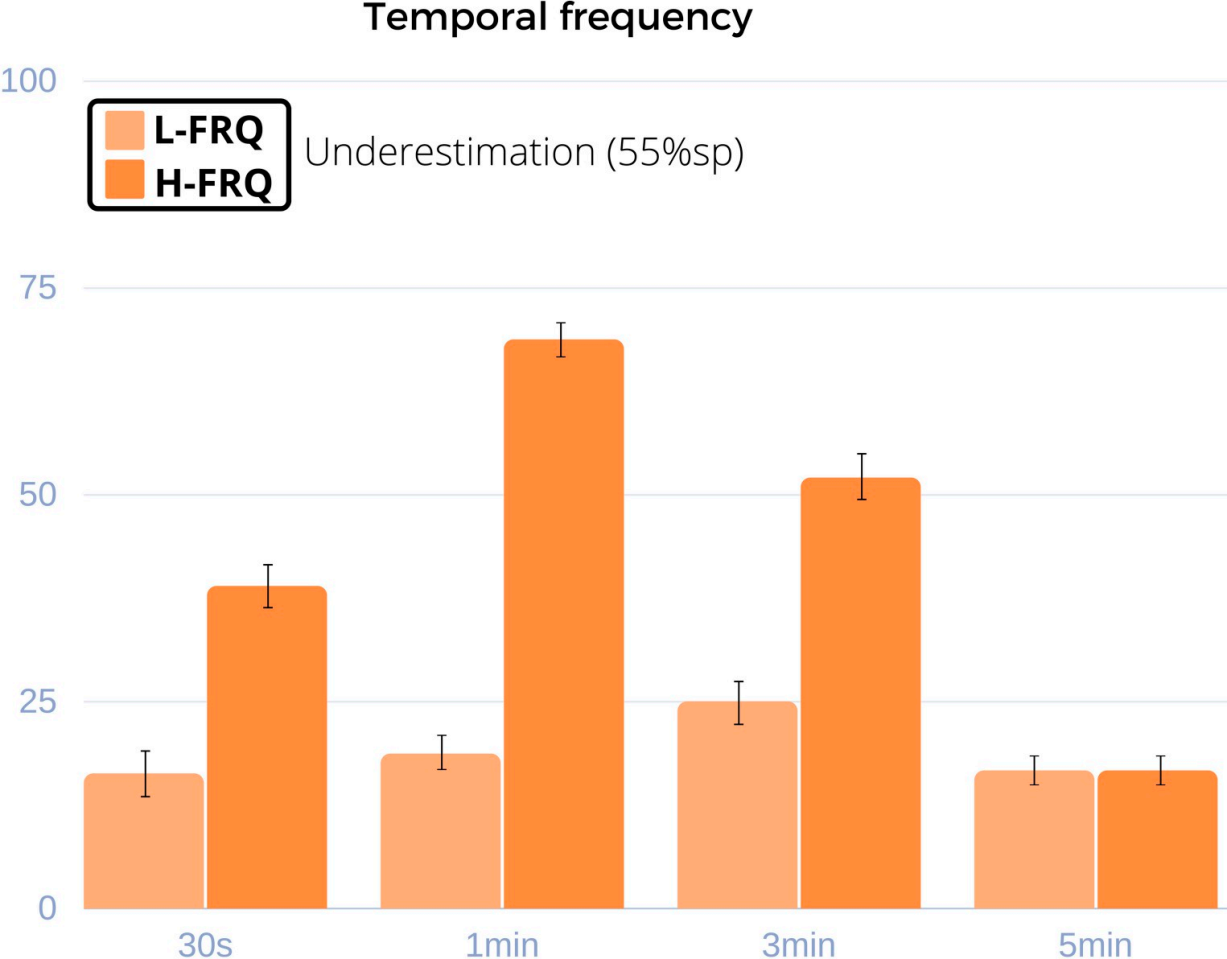
Results for Experiment 2. Y axis: % of answers. X axis: Temporal duration of the trials (30s, 1min, 3min, 5min). The displayed bars correspond to p_under,55_ at the 55% sp (underestimation). The percentage of correct answers for each case is complementary to the displayed value in the graph (p_corr,55_+p_under,55_ = 100%). Like in Experiment 1, underestimation is more frequent in high magnitude levels, in this case in trials with a duration of up to three minutes.

**Fig 6:**
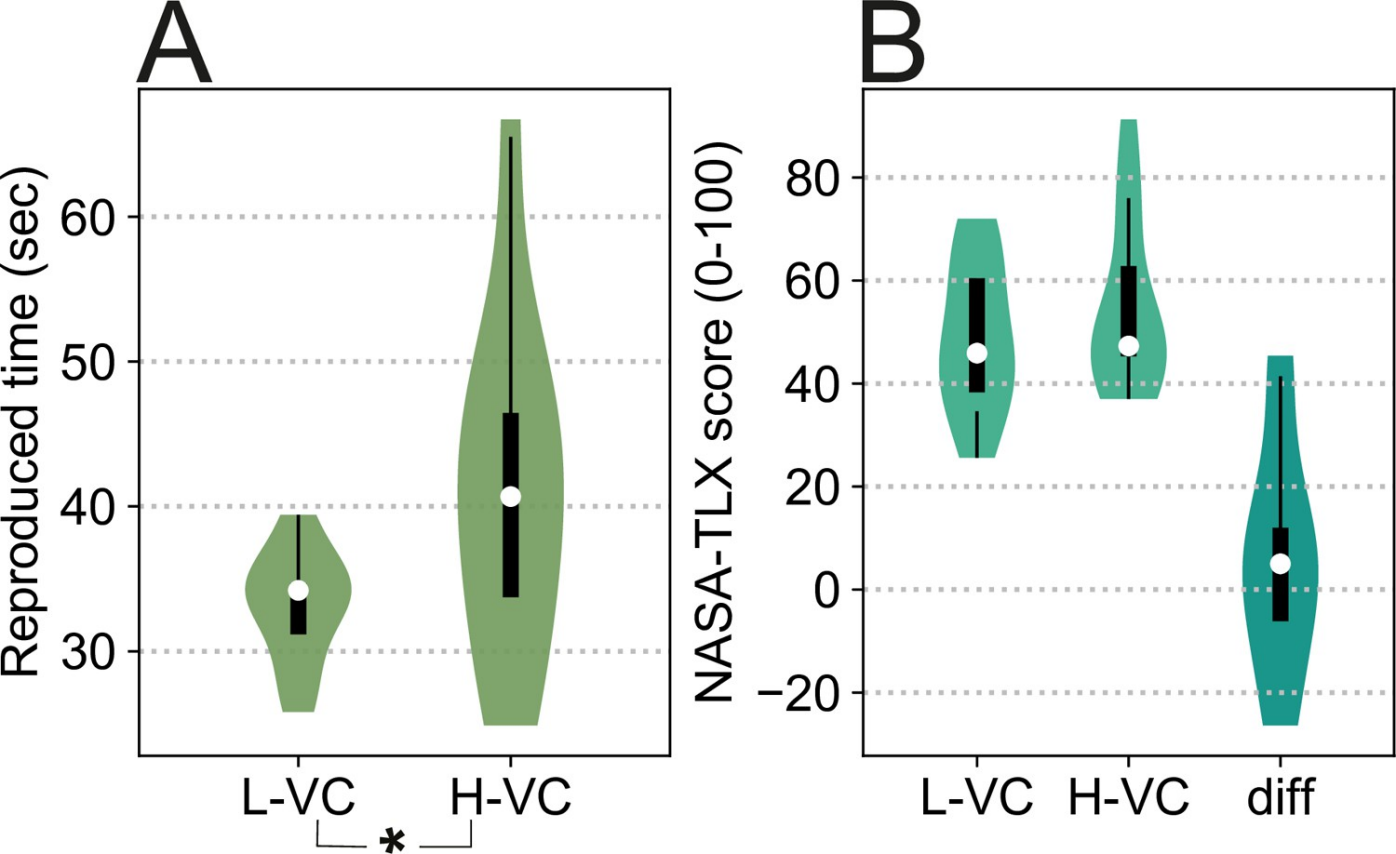
*Task B*: Experiment results. **A**: *Experiment 3*. Participants take longer to realize 30s have passed in H-VC, which suggests that time is compressed in the presence of larger perceived visual changes. **B**: NASA-TLX score (workload measure) for the visual complexity production tasks of each level. Right violin plot (“diff”) shows individual differences in NASA-TLX score (L-VC MINUS H-VC). Note that the negative values in the diff violin plot mean that for some users L-VC was experienced as more demanding than H-VC. Significant differences are marked with an asterisk.

In the following, we explain each of the experiments in detail. Table 1 presents a list of the acronyms used through this paper in order of occurrence. Our results can be found in Tables 2 and 3 and Figs 3–6. Our main experiment (Experiment 1) explores how different magnitudes of luminance contrast, temporal frequency and field of view affect half-duration judgements under a fixed viewing condition (static panoramas viewed in HMDs, Section 2.1). We conducted a follow-up replication in conventional displays which shows the same trend found in the work of Van der Ham et al. [9] (Section 2.2). Experiment 2 (Section 2.3) extends the studies from 30s to up to five minutes. Finally, Experiment 3 (Section 2.4) uses a different task design (duration judgements) to give an estimate of the extent to which temporal perception gets distorted with changes in the visual complexity of the stimuli.

**Table 1:** List of acronyms used through this work.

Acronym	Meaning
CD	Conventional displays
HMD	Head-mounted displays
VR	Virtual reality
FRQ	Frequency
CON	Contrast
FOV	Field of view
H-CON	High *magnitude* level of contrast
L-CON	Low *magnitude* level of contrast
HFM	Histogram flatness measure [21]
H-FRQ	High *magnitude* level of frequency
L-FRQ	Low *magnitude* level of frequency
H-FOV	High *magnitude* level of FOV
L-FOV	Low *magnitude* level of FOV
sp	Sampling point
p_over,45_	Percentage of “more than half” responses in the 45% sp (overestimation indicator)
p_under,55_	Percentage of “less than half” responses in the 55% sp (underestimation indicator)
p_corr,XX_	Percentage of correct answers in the XX% sp (accuracy indicator)
VC	Visual complexity
H-VC	High *magnitude* level of visual complexity
L-VC	Low *magnitude* level of visual complexity

**Table 2:** Results of Experiment 1. Accuracy (% of correct answers) is higher for high *magnitude* levels of contrast, frequency and FoV at 45% sp (“less than half” answers), while at 55% sp (“more than half” answers) it is always higher for low *magnitude* levels of contrast and frequency. At 45% sp, overestimation (incorrect responses, “more than half” answers) occurs more frequently for low *magnitude* levels. At 55% sp, underestimation (incorrect responses, “less than half” answers) occurs more frequently at high *magnitude* levels.

Factor	Sp	% of correct L/H (p_corr,45_ or p_corr,55_)	% of underestimated L/H (p_under,55_)	% of overestimated L/H (p_over,45_)
CON	45 (13.5s)	60.5(L)/**65.8(H)**	-	**39.5(L)**/34.2(H)
	55 (16.5s)	**57.9(L)**/42.8(H)	42.1(L)/**57.2(H)**	-
FRQ	45 (13.5s)	52.6(L)/**73.7(H)**	-	**47.4(L)**/26.3(H)
	55 (16.5s)	**58.2(L)**/48(H)	41.8(L)/**52(H)**	-
FOV	45 (13.5s)	62.8(L)/**68.5(H)**	-	**37.2(L)**/31.5(H)
	55 (16.5s)	**73.7(L)**/63.2(H)	26.3(L)/**36.8(H)**	-

**Table 3:** A) Results of the follow-up replication study with conventional displays. Accuracy (% of correct answers) is higher for high *magnitude* levels of contrast and frequency at 45% sp (“less than half” answers), while at 55% (“more than half” answers) it is always higher for low *magnitude* levels of contrast and frequency. At 45% sp, overestimation (incorrect responses, “more than half” answers) occurs more frequently for low *magnitude* levels. At 55% sp, underestimation (incorrect responses, “less than half” answers) occurs more frequently at high *magnitude* levels.

Factor	Sp	% of correct L/H (p_corr,45_ or p_corr,55_)	% of underestimated L/H (p_under,55_)	% of overestimated L/H (p_over,45_)
CON	45 (13.5s)	58.3(L)/**66.6(H)**	-	**41.7(L)**/33.4(H)
	55 (16.5s)	**62.9(L)**/42.7(H)	37.1(L)/**57.3(H)**	-
FRQ	45 (13.5s)	52.9(L)/**67.8(H)**	-	**47.1(L)**/32.2(H)
	55 (16.5s)	**61.7(L)**/32.6(H)	38.3(L)/**67.4(H)**	-

#### 2.1 Experiment 1 –Frequency, contrast, and field of view in HMDs

In *Experiment 1* we study how variations in the magnitude of the asemantic visual features *temporal frequency* (FRQ), *luminance contrast* (CON), and field of view (FoV) affect time perception while watching static panoramic imagery in head-mounted displays (HMDs). All the trials tested in this experiment had a duration of 30s. We denote as a *trial* any separable part of an experiment associated with an answer from the participant. *Experiment 1* was carried out using the study design we labeled *Task A*, which evaluates how changes in the magnitude of asemantic visual features affect time estimation, for a fixed viewing condition. Participants were first exposed to a sample trial duration to set the expectations. On each subsequent trial, participants were interrupted approximately halfway through the duration and asked to make a judgement about whether more or less than half of the full trial duration had elapsed [8]. We term these binary temporal judgements *half-duration judgements*. The binary task design has two advantages: first, we wanted participants to give us quick, intuitive answers. Second, compared to asking participants to directly estimate magnitudes (i.e., how much time has passed) binary judgements are less noisy and variable, at the expense of less information. The goal of *Task A* is different from the millisecond timing experiment design presented in the prestudies where participants compared the duration of *big* vs *small* stimuli in a single trial. Our goal with the prestudies was to replicate previous work in a different viewing condition (in this case, an HMD). For *Task A* we have tried to avoid memory-related confounding factors by presenting one single manipulation of a given visual feature in each trial.

*Participants and apparatus*. The stimuli were presented on an HTC Vive Focus, a portable HMD (2880x1600 spatial resolution, 75Hz, 110 visual degrees FoV). A total of 89 participants took part in *Experiment 1*. To avoid excessively large numbers of trials per participant leading to fatigue or learning effects, we split the visual features tested and participants into two groups. Group 1.1 consisted of 45 participants (20 female, mean age 28.7 years) who experienced *temporal frequency* x *luminance contrast* (low-level visual features) changes. Group 1.2 consisted of a different set of 44 participants (18 female, mean age 26.8 years) who experienced *FoV* (mid-level property) changes. All participants had normal or corrected-to-normal vision. All were naïve to the purpose and hypothesis of the study. These two affirmations hold for all the presented experiments.

*Stimuli*. We gathered a set of 420 panoramas from open-source web databases (Flickr, Unsplash, and Pixexid), all of them depicting natural indoor or outdoor scenes (see Fig 2). They were manually selected, excluding synthetic, cartoon, high-emotional valence (violence, parties, dramatic pictures, etc.) and written content. The stimuli were randomly arranged in sequences of 30 panoramas to compose each 30s experiment trial. Each image was shown for a total of 1.2s-1.8s, with a fade-in/fade-out effect of 0.5s between images, during which the images overlapped in order to create smooth transitions. Both the fading effect and variable image presentation durations were implemented to avoid the kind of predictable regularity that may facilitate counting. Instead of being presented for 1 second each, images in the sequence cycled through the following duration pattern: {0.4s, 0.2s, 0.4s, 0.6s, 0.8s, 0.6s}. Including the additional 0.5s transition between images, a group of six images had a total duration of 6 seconds. Five of these groups were sequenced to create the 30s trials.

*Luminance contrast*. Images or video sequences with a high *magnitude* level of contrast (H-CON) produce larger visual changes than those with a low *magnitude* level of contrast (L-CON). A representative sample of these stimuli can be found in Fig 2. We calculated the contrast of our 420 panoramas taking into consideration their most salient 2D crop spanning 45 by 65 degrees of visual angle [22], and using the histogram flatness measure (HFM) [21]. The computed contrast range of our full stimuli set was [0.17, 0.72]. We selected the 120 panoramas with least contrast with an L-CON in the range [0.17, 0.60], and 120 panoramas with the highest contrast with an H-CON in the range [0.60, 0.72]. The remaining 180 panoramas were used for the temporal frequency conditions.

*Temporal frequency*. We created the high and low temporal frequency conditions (H-FRQ and L-FRQ, respectively) out of the same stimulus set, to manipulate temporal frequency without changing the amount of visual information presented. Specifically, starting with the stimuli in the L-FRQ condition, we inserted three black frames per second to the entire image sequence to create a flickering effect in the H-FRQ condition.

*Field of view*. To reduce the differences in conditions to only the main factor under investigation, we similarly used the same stimulus set for both high and low FoV conditions (H-FOV and L-FOV, respectively). In this case, we used panorama images that were either shown in full size in the H-FOV condition or were reduced to their most salient [22] crops (2D re-projected regions of 45× 65 degrees of visual angle, Fig 2) in the L-FOV condition. H-FOV was designed to show visual changes in a larger portion of the visual field compared with L-FOV. Note that the entirety of Experiment 1 was carried out in HMDs to take advantage of the expanded field of view available.

*Procedure*. The experimental procedure was the same for both Groups 1.1 and 1.2, using half-duration judgements for measuring the perception of time (*Task A*, Fig 1). Before beginning *Experiment 1*, participants were explicitly told not to count to estimate time passage. At the beginning of the experiment, they were informed of the duration of each of the trials (30s), and provided with a 30s sample trial, which was interrupted with on-screen message to denote when half of the duration (15s) had elapsed. After the sample trial, the experiment proceeded with a set of consecutive 30s trials. Within each trial, participants would be prompted to make a half-duration judgement at a given (unknown to participants) sampling point (sp) of 45% or 55% of the total trial duration (i.e., at 13.5s or 16.5s in a 30s interval): ***Has more or less than half of the time elapsed*?** Participants answered using the HMD controllers. They were instructed to answer as fast as possible, and the trial continued automatically after their response. At the end of each trial, an additional binary question appeared: ***Do you think your previous answer was correct*?** Participants again answered using the HMD controllers. Participants in Group 1.1 completed a total of 32 trials, for a total experiment duration of approximately 20 minutes. Participants were asked to make a half-duration judgement at a sampling point of 45% for half the trials, and at a sampling point of 55% for the other half. The trials were randomly ordered for each participant. Across trials for a given participant the magnitude level (high vs low) and the feature chosen (contrast vs frequency) could vary. However, within any particular trial, all the stimuli were consistent in magnitude and feature manipulation. As a reminder, the goal was to accumulate the effects of a particular manipulation over the full duration of a trial (in this case 30s) to evaluate how the manipulation affects time perception at 30s intervals. Similar to Group 1.1, participants in Group 1.2 completed a total of 8 trials, for a total experiment duration of approximately 5 minutes with the difference being a manipulation in the sampling point and magnitude of the FoV condition for this group.

*Statistical analysis*. A 2 magnitude levels (high vs low) ×2 visual features (contrast vs frequency) ×2 sampling points (45% vs 55%) ANOVA was used to check for significant differences for the 45 participants (Group 1.1) who experienced luminance contrast and temporal frequency manipulations. A 2 (FOV levels: high vs low) ×2 (sampling points) ANOVA was used to check for significant differences for the 44 participants (Group 1.2) who experienced the FoV manipulation. The answer variable was binary (“more than half” or “less than half” of the time elapsed) in both analyses. *Post hoc* analyses for this experiment can be found in the Appendix A in S1 File. All statistical analyses were carried out using Matlab. ANOVA post-hoc power was calculated with additional scripts [23]. Effect sizes were calculated using Harald’s Toolbox for Matlab [24] (partial eta squared for ANOVA). Additionally, we analyzed Groups 1.1 and 1.2 together (89 participants) with a GLMM to check for interactions of the different visual features for completeness. The information of the GLMM analysis can be found in the Appendix B in S1 File.

*Results*. We measure and analyze: (i) the percentage of “less than half” responses in the 55% sp with respect to the total number of responses for this sampling point, which is an indicator that time felt shorter (or was underestimated) according to the half-duration judgement (p_under,55_); and (ii) the percentage of “more than half” responses in the 45% sp with respect to the total number of responses for such sp (p_over,45_), an indicator that time felt longer (or was overestimated). These two values are complementary to the percentage of correct responses (p_corr,45_ and p_corr,55_) with respect to the total number of responses, such that, for any given condition, p_corr,45_+p_over,45_ = 100% and p_corr,55_+p_under,55_ = 100%. In *Experiment 1*, underestimation was more common for H-CON while overestimation was more common for L-CON. Analogously, underestimation was more common for H-FRQ and H-FOV and overestimation was more common for L-FRQ and L-FOV (see Table 1 and Fig 3). With a significance level established at p = 0.05 and power of 0.895 and 0.894, both ANOVAs revealed that *magnitude* had a significant effect on the answers (F = 45.03, p<0.001, partial η2 = 0.580 for the three-way ANOVA, F = 12.39, p<0.001, partial η2 = 0.228 for the two-way ANOVA), while the sampling point (F = 0.91, p = 0.340, partial η2 = 0.012 for the three-way ANOVA; F = 0.49, p = 0.482, partial η2 = 0.009 for the two-way ANOVA) and *visual features* (F<0.01, p = 0.964, partial η2<0.001 only tested in the three-way ANOVA for frequency and contrast) did not. The additional GLMM analysis yielded consistent results with the separated ANOVAs, with only the magnitude factor having a significant effect on the response variable (t = -3.7641, CI {-1.142, -0.360}, p<0.001). We observe a similar effect for high magnitude levels across the three visual features: H-CON, H-FRQ, and H-FOV all make time seem shorter compared to their low magnitude counterparts. Moreover, participants were fairly confident in their answers, as inferred from the binary responses at the end of each trial, where participants indicated if they agreed with their half-duration judgement in retrospect, after the whole interval had elapsed. We define a trial as rectified if participants believe at the end of a trial that their previous answer was wrong. *Experiment 1* had a rectification percentage of less than 5%.

#### 2.2 Follow-up replication in conventional displays

The work of Van der Ham et al. [9] suggests that similar half-duration judgements can be elicited with HMDs and conventional displays (CDs) if viewing conditions are similar. Our prestudies follow the same trend. In Experiment 1, we addressed *interval* timing, with experiments done on HMDs only. Thus, we ran a follow-up study to replicate part of Experiment 1 on CDs. We replicated Experiment 1, but focused our attention on the two low-level visual features that should ideally generalize across display types: luminance contrast (CON) and temporal frequency (FRQ).

*Participants and apparatus*. The stimuli were presented on a Samsung display (S24F350FHU, 1920x1080 spatial resolution, 60Hz) at a distance of 60cm from the viewer. Seven participants completed the follow-up study (3 female, mean age 22.4 years).

*Stimuli*. The stimuli used in this study consisted of a set of 420 images of natural indoor and outdoor scenes from open-source web databases with a CC license (Flickr, Unsplash, and Pixexid) depicting natural indoor and outdoor scenes. These were selected following the same exclusion criteria as in Experiment 1. The presentation of the images, duration, fade-in/fade-out effects, etc., were the same as in Experiment 1, yielding test trials that included 30 images and were 30s long. Participants completed a total of 16 trials. The duration of the experiment was 10 minutes.

*Luminance contrast and temporal frequency*. The different magnitude levels of CON and FRQ were achieved following the same procedure as in *Experiment 1*. The computed contrast range (HFM, see Section 2.1) of our full stimuli set was [0.55, 0.84]. We selected the 120 images with least contrast with an L-CON in the range [0.55, 0.64], and 120 images with the highest contrast with an H-CON in the range [0.71, 0.84]. The remaining 180 images were used for the temporal frequency conditions.

*Procedure*. This study was carried out following the same procedure described in *Experiment 1*, where participants indicated whether more or less than half of the time had elapsed, with the sole difference that instead of wearing an HMD and using its controllers as the input device, in this version of the study participants were entering their responses on a keyboard while looking at the CD. In this study, each participant experienced 8 conditions: 2 magnitude levels (high vs low)×2 visual features (contrast vs frequency)×2 sampling points (45% vs 55%).

*Statistical analysis*. A 2×2×2 ANOVA was used to check for significant effects (with *visual feature*, *magnitude* and *sampling point* as factors). The answer variable was binary (“more than half” or “less than half” of the time elapsed). *Post hoc* analyses for this experiment can be found in the Appendix A in S1 File.

*Results*. Using the same measures described in *Experiment 1*, we found similar tendencies in time perception between HMDs and CDs. Table 3 and Fig 4 show the results of this study. The underestimation and overestimation trends follow those found in *Experiment 1*: H-CON and H-FRQ elicited higher underestimation rates while overestimation was more frequent for L-CON and L-FRQ. However, while the trend observed is the same, no significant effect was found for any of the tested factors *(magnitude* F = 0.56, p = 0.454; *visual feature* F = 0.56, p = 0.454; *sampling point* F = 1.27, p = 0.263).

#### 2.3 Experiment 2 –Extended durations up to five minutes

To further analyze the stability of the observed time compression effect over extended periods, we tested temporal frequency (FRQ) effects at trials of longer duration. We only considered temporal frequency as a visual feature in Experiment 2 to keep the size of the experiment tractable, since we found no significant differences between the tested *visual features* in *Experiment 1*. To maintain participant engagement and for ecological validity at longer durations, *Experiment 2* employed sequences of short video clips arranged into “movies”, instead of static images. The trials tested in *Experiment 2* varied in duration, including: 30s, one minute, three minutes, and five minutes.

*Participants and apparatus*. In the follow-up replication study (Section 2.2) we verified that similar results could be found both in HMDs and CDs when using analogous experimental set ups. Given that Experiment 2 deals with longer temporal durations, to avoid a potential confounding effect of fatigue caused by prolonged exposure [25] in HMDs, we use CDs. The stimuli were presented on a Samsung display (S24F350FHU, 1920x1080 spatial resolution, 60Hz), at a distance of 60cm from the viewer. Fifty-one participants took part in *Experiment 2* (20 female, mean age 22.9 years).

*Stimuli*. The videos used in *Experiment 2* consisted of 700 three-second video clips from the Moments dataset [26]. The set of videos was manually curated to exclude high arousal actions, synthetic content, text, or manipulated playback speed. Randomly ordered sequences of videos were arranged to form each trial. Since video clips had a fixed duration of 3s, the number of video clips per trial was a function of the length of the trial (10 clips for 30s trials, 20 clips for one minute trials, 60 clips for three minute trials and 100 clips for five minute trials). No transition effects were applied between different clips inside a given trial. Videos were played as continuous “movies” composed of back-to-back 3s clips.

*Temporal frequency*. To induce a high temporal frequency in sequences of videos, we subdivided each 3s clip into 1s cuts that were then reshuffled, effectively increasing temporal changes in visual content without changing the totality of visual information presented.

*Procedure*. *Experiment 2* was carried out using half-duration judgements for measuring the perception of time, following the procedure outlined in *Experiment 1* (see Section 2.1). Participants were randomly assigned to one of the four possible duration conditions, and completed all the trials with the same presentation duration to avoid bias effects due to differences in trial durations. For simplicity, we only used the 55% sampling point (prompting participants to make temporal judgements after 16.5s elapsed within 30s trials, after 33s for one minute trials, 99s for three minute trials and 165s for five minute trials), which means each duration had only two possible conditions (2 magnitude levels x 1 sampling point x 1 visual feature). Each participant completed 6 trials, for a total duration between three minutes (in the case of 30s trials) and 30 minutes (for five minute trials).

*Statistical analysis*. Each of the sampled trial durations was analyzed separately, testing for significant differences in high vs low *magnitude* levels of FRQ with Chi-square proportions tests. The answer variable was binary, as in *Experiment 1*.

*Results*. With a significance level established at p = 0.05 the duration of H-FRQ stimuli was significantly underestimated for trial durations up to three minutes (higher p_under,55_ for H-FRQ, see Fig 5): 16.3% of trials in L-FRQ condition vs. 38.9% in H-FRQ at *30s* (χ^2^ = 13.27, p = 0.001, *post hoc* power = 0.83, ES = 0.586); 18.7% L-FRQ vs 68.7% H-FRQ at *one-minute* (χ^2^ = 50.99, p = 0.001, *post hoc* power = 0.99, ES = 0.635); 25% L-FRQ vs. 52% H-FRQ at *three-minute* trials (χ^2^ = 15.39, p = 0.001, *post hoc* power = 0.88, ES = 0.579). At *five-minut*e trials, however, the effect was no longer present: 16.6% L-FRQ vs. 16.6% H-FRQ (χ^2^ = 0, p = 1).

#### 2.4 Experiment 3 –Quantification of the perceived temporal distortion

All the previous experiments in the paper used half-duration judgements, collected as binary responses (*Task A* study design) to evaluate the effects of changes to visual features on the perception of time. Experiments 1–2 confirm that the perception of time can indeed be distorted by manipulating the magnitude of a visual feature (e.g., high vs low contrast, frequency, etc.). *Experiment 3* was then designed to give an estimate of the extent to which temporal perception gets distorted, by using traditional duration judgements. In this case, rather than making a binary half-duration judgement, participants produce a numerical estimate of the elapsed duration. In *Experiment 1* we found that only differences in *magnitude* of the different visual features had a significant effect on time perception. However, we did not find any significant difference between the three tested visual features: frequency, contrast and field of view. Since all the features were equally effective, for *Experiment 3* we focused on a fourth, more abstract, feature: visual complexity. We define *visual complexity* as the number of distinct sources of visual content inside a given scene. Visual complexity was chosen as a proxy of real-world tasks that require simultaneous attention to multiple screens or sources of content. Visual complexity uses the principle common to all the previously tested features, whereby high magnitude levels of the feature trigger larger spatiotemporal per-pixel variations than their lower magnitude counterpart. In summary, in *Experiment 3* we estimate how much magnitude variations of *visual complexity* (VC) affect time perception. All the trials tested in this experiment had a target duration of 30s.

*Participants and apparatus*. The stimuli were presented on an Oculus Rift CV1 HMD (2160x1200 spatial resolution, 90Hz, 110 visual degrees FoV). Eleven participants took part in *Experiment 3* (5 female, mean age 25.2 years).

*Stimuli*. The same set of videos described in *Experiment 2* were used in this experiment. In the low visual complexity (L-VC) condition, four screens inside the virtual scene displayed the same identical video simultaneously. In the high visual complexity (H-VC) condition, each screen displayed a different video, effectively augmenting the sources of visual information inside the scene, as well as the spatial changes of the visual stimuli.

*Procedure*. *Experiment 3* was carried out using *Task B*: a double production task [27]. *Task B* was designed to study the magnitude of time estimation errors under larger visual changes (Fig 1). At the beginning of the experiment, participants were explicitly instructed not to count to get a feeling of time passing, like in *Task A*. Participants were first exposed to an *empty* virtual scene for 30s: a gray background and a fixation cross surrounded by a lighter gray circle of two degrees of visual angle situated in the middle. During these 30s, an **auxiliary task** was performed in order to prevent participants from counting. Auxiliary tasks help maintain engagement without significantly increasing cognitive load. The letters “A” and “L” would appear at a fixed position to the left or right of the fixation cross, respectively. When participants saw one of those letters in the scene, they had to press the corresponding key on a keyboard as fast as possible. A/L appeared at random intervals of 0.5-2s, 1.5° visual angle to the left or right of the fixation cross, and with a vertical size of 100 pixels. When the 30s had elapsed, and after a three-second gap, the circle around the fixation cross turned green and the **empty production** subtask started: participants had to press a key to indicate when 30 more seconds had elapsed, while continuing with the auxiliary task. After completing the empty production task, participants moved on to the next task after a keypress. Empty production tasks were used to measure individual baseline estimation in the absence of a stimulus. Participants were then shown a virtual empty room with four flat screens displaying videos on one of its walls. The **visual complexity production** subtask started: across both H-VC and L-VC conditions, participants had to indicate when 30s had elapsed by pressing a key. At the same time, they had to complete a new auxiliary task: pressing a key when something red appeared in any of the videos. Like in the first auxiliary task, the red detection task was designed with the intention of increasing engagement through the experiment and preventing users from explicitly counting. Immediately after the visual complexity production, participants completed a NASA-TLX questionnaire [28] to measure the cognitive load elicited by the visual complexity production subtask. We used a within-subjects design: each participant completed this experimental sequence twice, once with H-VC, once with L-VC, but with a different set of stimuli. The order of the conditions (H-VC and L-VC) was randomized to avoid ordering effects. Finally, each participant completed an additional empty production subtask, for a total of three empty productions, from which a robust individual measure of production accuracy was obtained by averaging.

*Statistical analysis*. An ANOVA was carried out to compare differences in *magnitude* for H-VC and L-VC levels. NASA-TLX questionnaire differences were tested with a t-test. The answer variables were continuous (for the produced durations) or discrete (for the NASA-TLX scores). The independent variable was binary (for low and high magnitude levels of visual complexity). *Post hoc* analyses for this experiment can be found in the Appendix A in S1 File.

*Results*. The mean ratio of empty productions to ground truth duration (target duration of 30s) was 1.12, which suggests a small overestimation of time in the baseline condition. To account for individual differences in time estimation, we computed a ratio between the visual complexity production and the empty production on a per-participant basis. We fixed the empty productions as the baselines for each participant. Mean accuracy in the auxiliary task associated with the empty production (A/L detection) was 99.43%. Mean accuracy (correct keypresses divided by the total number of keypresses) in the second auxiliary task (red detection) was 99.13% (true positive rate) with only 4.15% of detection failures (false positive rate, i.e., cases in which a red object appeared on screen but there was no keypress). The mean response time was 0.416s for the auxiliary tasks. Participants took more time to indicate that 30s had passed in the H-VC condition, suggesting that time was perceived as significantly shorter under higher visual complexity (ratio of 1.38 for H-VC vs 1.10 for L-VC, power = 0.407, F = 4.98, p = 0.0372, partial η2 = 0.24, normality of distribution checked with Anderson-Darling tests). In other words, participants took, on average, 25.4% longer to perceive that 30s had passed in H-VC than in L-VC. Fig 6 illustrates this difference.

Additionally, participants completed a NASA-TLX questionnaire after each visual complexity production to provide a measure of cognitive load differences between the H-VC and L-VC condition levels. There were no significant differences in perceived workload between H-VC and L-VC productions (54.85 mean score for H-VC, 48.83 for L-VC, normal distribution checked with Anderson-Darling tests, t-test t(10) = 0.8857 p = 0.397, Fig 6).

## Discussion

Our series of experiments reveal consistent trends in how asemantic visual features directly alter half-duration judgements on prospective interval timing paradigms. Our results suggest that the perception of time is compressed in the presence of larger spatiotemporal visual changes (elicited in high *magnitude* levels of each visual feature) and dilated when smaller spatiotemporal visual changes are perceived. Moreover, participants felt confident about their half-duration judgements, as evidenced by the fact that they did not change their answers at the end of each trial (less than 5% rectification rates on average). Our findings hold true for different types of visual changes, and different timing intervals, up to and including three minutes. Debriefing sessions with participants in *Experiment 2* indicated that confounding factors like fatigue effects might be masking the main effect of visual features in the longer five-minute trials, requiring further investigation.

*Experiments 1* and *2* relied on a binary task (*Task A*) to investigate the existence of a relationship between the magnitude of visual features and half-duration judgements. In *Experiment 3*, we used duration judgements to estimate *how much* time perception could be compressed or dilated using a double production task (*Task B*) and the more abstract feature of visual complexity. In *Experiment 3* the ratio of the *empty* productions (those done in the absence of visual stimuli) to the target 30s duration was 1.12. This ratio was consistent with previous literature [5], which estimated a mean 10% deviation from actual time in interval timing judgements. Following this same trend, both H-VC and L-VC made time be perceived as shorter when compared to the empty production (participants took longer to indicate 30s had passed) since both conditions contain more visual changes than an empty scene. Despite the change in task design, we observed the same trend as in *Task A*, where larger visual changes perceptually compressed time. The high accuracy achieved on the auxiliary tasks in *Experiment 3* suggests that participants were engaged throughout the experiment and concentrated on task completion when 30s trials were presented. It would be highly unlikely for participants to accurately complete these tasks while also counting, since their working memory capacity is limited [29]. Participants also self-reported, in a post-study questionnaire, that they were not counting, so we have good reason to believe that we are indeed measuring perceived estimates of time. However, *Experiment 3* had a low power (0.407, below the commonly accepted threshold of 0.8). This means that the interpretation of *Experiment 3* results, while interesting, is limited due to a small sample size. Further experiments should be carried out to confirm the preliminary effects found in this work. The fact that we found no significant differences between the perceived workload of the different conditions (H-VC vs L-VC) in *Experiment 3* suggests that participants did not experience more cognitive demand in either condition. We believe our findings through Experiments 1–3 cannot be simply attributed to an increase in cognitive load due to the larger per-pixel variations (spatiotemporal visual changes) that participants have to process in the high magnitude levels of each visual feature. Instead, our results could be explained by demands on attentional resources as discussed below.

In retrospective judgments, participants are not informed in advance that a temporal judgement will be made [8], and as a result have to rely on memory in estimating the passage of time, which may result in confounds. Instead, we focused on the real-time perspective with stimuli that more closely approximate real-world applications and prospective judgements where people are aware of the passage of time. Contrary to findings for millisecond timing [5, 6], we found that larger asemantic visual changes actually shorten perceived interval time perception in a prospective setting. Our experiments suggest that all of the following visual features affect time perception: spatiotemporal visual changes (luminance contrast and temporal frequency), field of view, and the number of distinct sources of visual information (visual complexity, which in our case was the number of unique video streams). This apparent inversion of the effect when compared with millisecond timing might be explained by the fact that different timing mechanisms may guide the perception of different temporal magnitudes [5].

The perceived compression of time under large visual changes is the inverse of the well-known oddball effect [30]. In the oddball effect, no visual changes (a repetition of the same stimuli) cause time to be perceived as shorter when compared with a visual change (a different stimulus). However, this might be more related to prior expectations than to visual changes. In the oddball effect, users *get used to* watching the same stimulus repeatedly. The appearance of an unexpected, different stimulus contradicts the users’ expectations, potentially *capturing their attention* [31]. In contrast, our experiments are designed to prevent participants from becoming accustomed to a particular duration or condition, for several reasons: first, the order in which trials from different magnitudes or visual features are presented to the participants is randomized. Second, we evaluate the answer of our participants at two possible sampling points of which they are unaware during the experiment, to prevent them from learning the moment at which the question presented in each trial will appear. Third, we make use of realistic, distinct stimuli for each trial in order to maintain participant engagement, which we believe prevents participants from forming specific expectations.

The stimulus information processing load is a contextual factor that can inversely affect duration judgements in prospective paradigms [32]. Although low-level visual processing might involve autonomous processes that do not need the support of cognitive resources, high order cognitive processes and early perceptual processing may not be completely independent [33]. In that sense, an increase in visual processing due to larger visual changes could be affecting timing interval mechanisms, either via an increased use of cognitive resources, which are limited, or by a shift in attention from the temporal task to the observation of the scene around the participant. A possible explanation for our findings could be found in the attentional resources theory [34]. In this work, Brown studied how nontemporal tasks affected a concurrent temporal task. The main findings showed a classic interference effect: “*the concurrent nontemporal tasks caused temporal productions to become longer (longer productions represent a shortening of perceived time) and/or more variable than did timing-only conditions*”. Under the attentional resources theory, a limited amount of attentional resources are split between the tasks being carried out at each moment. Nontemporal tasks thus take resources away from the attention that would be otherwise allocated to the feeling of time passing. In our case, the higher volume of visual information (larger perceived visual changes) may distract attention from the passage of time, resulting in time distortions. This change in the focus of attention from temporal to nontemporal tasks is known to have a major influence on timing behavior according to the *attentional-gate model* [32] and related empirical studies [35]. In the attentional-gate model, allocation of attention to time acts like a gate that regulates how often or how much of the pulses produced by a temporal pacemaker are then cognitively processed. In our case, the high levels of the different visual features might elicit a shift of attention from temporal to visual processing, causing fewer temporal pulses to be processed and effectively shortening the perceived time. Conversely, low levels of our visual features might require less processing (thus attention could be shifted to temporal processing). This low attentional demand could even cause a feeling of boredom in comparison [36], which would in turn lengthen the perceived time. In fact, this feeling of boredom might be one of the reasons why we could not directly extend the experiment of Xuan et al. [6] from millisecond to interval timing in our prestudies: our participants did not have enough information to process and the different conditions were not making up for that lack of stimulation.

### Limitations

Throughout our experiments, due to circumstances beyond our control, we had to adapt to a change in HMD hardware between Experiments 1 and 3, and a limited participant pool in the follow-up study to Experiment 1 (Section 2.2) and in Experiment 3. While the hardware (the HMDs) used in Experiment 1 (HTC Vive Focus) and Experiment 3 (Oculus HMD CV1) was different, we were careful to ensure that both HMDs shared the same key characteristics: both HMDs have spatial (6DoF) tracking, the same FOV (110 visual degrees) with a very similar refresh rate (75Hz for the Focus and 80Hz for the Oculus). Besides, the resolution of the stimuli displayed in both Experiments was the same, regardless of the native HMD resolution. We thus believe that this change did not have an effect on the answers of our participants. For this paper, we derive our main conclusions mostly from the analyses of Experiment 1, which was run on 89 participants and had sufficient statistical power. For completeness, we chose to nevertheless report the results from the follow-up study to Experiment 1 and Experiment 3, despite those experiments having low power. While we do not base our conclusions on these experiments, they do provide further validation that our observed trends from Experiment 1 continue to hold under changing conditions.

### Future directions

It has also been reported that aging affects time perception [37], with older adults experiencing perceptual time compression. To study how our reported effects generalize across demographic variables such as age, it would be necessary to expand the current participant pool (with an average age of 25.31 years; SD: 4.49). Moreover, we designed the experiments to ensure that higher *magnitude* levels of each visual feature induced larger visual changes across time. However, high *magnitude* levels also implied higher absolute values of each studied visual feature. Evidenced by Montague’s theory [20] and the *perceptual break* we found in our pilot studies when trying to directly extend an experiment with simple, fixed stimuli to interval timing, we believe that the changes in time perception are caused by larger visual changes being perceived, instead of by the absolute visual value. Nevertheless, a full isolation of the magnitude change from its absolute value could help confirm this in future work. Furthermore, we only sampled two sparse levels (low and high) for the magnitude of each visual feature. A more fine-grained sampling strategy with an increased number of magnitude levels could further illuminate the observed effects in an analytical manner. Another interesting question for future investigation is the minimum required change in visual stimuli to observe differences in time perception. Our low and high levels for each visual feature were selected based on preliminary tests, but different variations between these levels might result in differently sized effects.

### Conclusion

In summary, we suggest that real-world, interval-scale time perception can be compressed or dilated through asemantic and realistic visual changes. These findings imply that we can alter time perception without affecting the semantic content, making these results practical for application purposes. When designing applications where time judgements are relevant, our findings could be applied as general design guidelines: i.e., use a high contrast color palette, show changes in a larger portion of the field of view, or make faster camera cuts in a movie scene to make time seem shorter. Related to HMDs, compressing perceived time might also be helpful when controlling fatigue [25]. These findings have the potential for profound impact on practical applications, such as reducing perceived discomfort during medical treatment with virtual reality immersion [1], improving response time in highly dynamic tasks such as vehicle driving with heads-up displays, or lowering fatigue in professional training [4].

## Supporting information

S1 Movie Example of tasks A and B experimental flow. This video shows how tasks A and B are performed using a headset. For shortness, the video is accelerated.
(MP4)

S1 File Appendices A and B: Additional statistical analyses and post-hoc studies of the experiments described in this work.
(DOCX)
