# Supplementary material for: Larger visual changes compress time: The inverted effect of asemantic visual features on interval time perception
Source: PLoS One. 2022 Mar 22;17(3):e0265591. doi: 10.1371/journal.pone.0265591 (PMC8939824; doi:10.1371/journal.pone.0265591)
Supplement: S1 File — Appendices A and B: Additional statistical analyses and post-hoc studies of the experiments described in this work. (DOCX) [file pone.0265591.s002.docx]

Larger visual changes compress time: The inverted effect of asemantic visual features on interval time perception

**Authors**

Sandra Malpica,^1,*^ Belen Masia,^1^ Laura Herman^2^, Gordon Wetzstein^3^, David M. Eagleman^4^, Diego Gutierrez^1^, Zoya Bylinskii^2^, and Qi Sun^5,2,*^

**Affiliations**

^1^Universidad de Zaragoza, I3A

^2^Adobe, Inc.

^3^Department of Electrical Engineering, Stanford University

^4^Department of Psychiatry, Stanford University School of Medicine

^5^New York University

*To whom correspondence should be addressed. E-mail: [qisun@nyu.edu](mailto:qisun@nyu.edu) (QS) or [smalpica@unizar.es](mailto:smalpica@unizar.es) (SM)

**Abstract**

Time perception is fluid and affected by manipulations to visual inputs. Previous literature shows that changes to low-level visual properties alter time judgments at the millisecond-level. At longer intervals, in the span of seconds and minutes, high-level cognitive effects (e.g., emotions, memories) elicited by visual inputs affect time perception, but these effects are confounded with semantic information in these inputs, and are therefore challenging to measure and control. In this work, we investigate the effect of asemantic visual properties (pure visual features devoid of emotional or semantic value) on interval time perception. Our experiments were conducted with binary and production tasks in both conventional and head-mounted displays, testing the effects of four different visual features (spatial luminance contrast, temporal frequency, field of view, and visual complexity). Our results reveal a consistent pattern: larger visual changes all shorten perceived time in intervals of up to 3min, remarkably contrary to their effect on millisecond-level perception. Our findings may help alter participants' time perception, which can have broad real-world implications.

**Appendix A – ANOVA *post hoc* tests.**

In the following, we report the *post hoc* tests of our ANOVAs, including a visualization of the multiple comparison of population marginal means.

**Experiment 1.**

**Statistical Analysis.** A 2$\times$2$\times$2 ANOVA was used to check for significant effects (with *magnitude, visual feature (frequency and contrast),* and *sampling point* as factors) for Group 1.1 (45 participants). A 2$\times$2 ANOVA was used to check for significant effects (with *magnitude* differences in the FoV and *sampling point* as factors) for Group 1.2 (44 participants). The answer variable was binary (“more than half” or “less than half” of the time elapsed) in both analyses. With a significance level established at p=0.05 and power of 0.895 and 0.894, both ANOVAs revealed that *magnitude* had a significant effect on the answers (F=45.03, p<0.001, partial η2=0.580 for the three-way ANOVA, F=12.39, p<0.001, partial η2=0.228 for the two-way ANOVA), while the sampling point (F=0.91, p=0.340, partial η2=0.012 for the three-way ANOVA; F=0.49, p=0.482, partial η2=0.009 for the two-way ANOVA) and *visual features* (F<0.01, p=0.964, partial η2<0.001 only tested in the three-way ANOVA for frequency and contrast) did not. No significant interactions between the fixed factors were found. Tables A1 and A2 show each ANOVA in detail, while Figures A1 and A2 show the post-hoc visualization: each pairwise comparison between the possible different conditions where only groups with different magnitude were significantly different.

| Source | Sum Sq. | D.F. | Mean Sq. | F | P-value |
| --- | --- | --- | --- | --- | --- |
| Magnitude | 10.936 | 1 | 10.936 | 45.029 | <0.001 |
| Sampling point | 0.221 | 1 | 0.221 | 0.992 | 0.340 |
| Visual Feature | <0.001 | 1 | <0.001 | 0.002 | 0.964 |
| userID | 7.542 | 44 | 0.1714 | 0.706 | 0.927 |
| magnitude*sp | 0.003 | 1 | 0.003 | 0.013 | 0.908 |
| magnitude*visFeat | 0.059 | 1 | 0.059 | 0.241 | 0.624 |
| sp*visFeat | 0.001 | 1 | 0.001 | 0.005 | 0.943 |
| magnitude*sp*visFeat | 0.089 | 1 | 0.089 | 0.368 | 0.544 |
| Error | 338.059 | 1392 | 0.243 | - | - |
| Total | 356.996 | 1443 | - | - | - |

**Table A1**. 2$\times$2$\times$2 ANOVA of Experiment 1 (Group 1.1), with fixed factors: magnitude, sampling point and visual feature. The effect of each participant was considered as a random variable (userID, underlined). First and second order interactions between the fixed variables were considered. Only *magnitude* had a significant effect in the response variable.

| Source | Sum Sq. | D.F. | Mean Sq. | F | P-value |
| --- | --- | --- | --- | --- | --- |
| Magnitude | 2.774 | 1 | 2.774 | 12.390 | <0.001 |
| Sampling point | 0.111 | 1 | 0.111 | 0.496 | 0.482 |
| userID | 9.275 | 43 | 0.216 | 0.963 | 0.541 |
| magnitude*sp | 0.392 | 1 | 0.392 | 1.752 | 0.187 |
| Error | 68.293 | 305 | 0.224 | - | - |
| Total | 80.845 | 351 | - | - | - |

**Table A2**. 2$\times$2 ANOVA of Experiment 1 (Group 1.2), with fixed factors: magnitude and sampling point. The effect of each participant was considered as a random variable (userID, underlined). First order interactions between the fixed variables were considered. Only *magnitude* had a significant effect in the response variable.

**Figure A1. *Post hoc* analysis of the three-way ANOVA (Contrast and Frequency conditions).** Significant differences were found only between groups with different magnitude levels. Figure A1 shows the 95% confidence interval of the mean difference between each multiple comparison, as well as the p-value for each comparison.

**
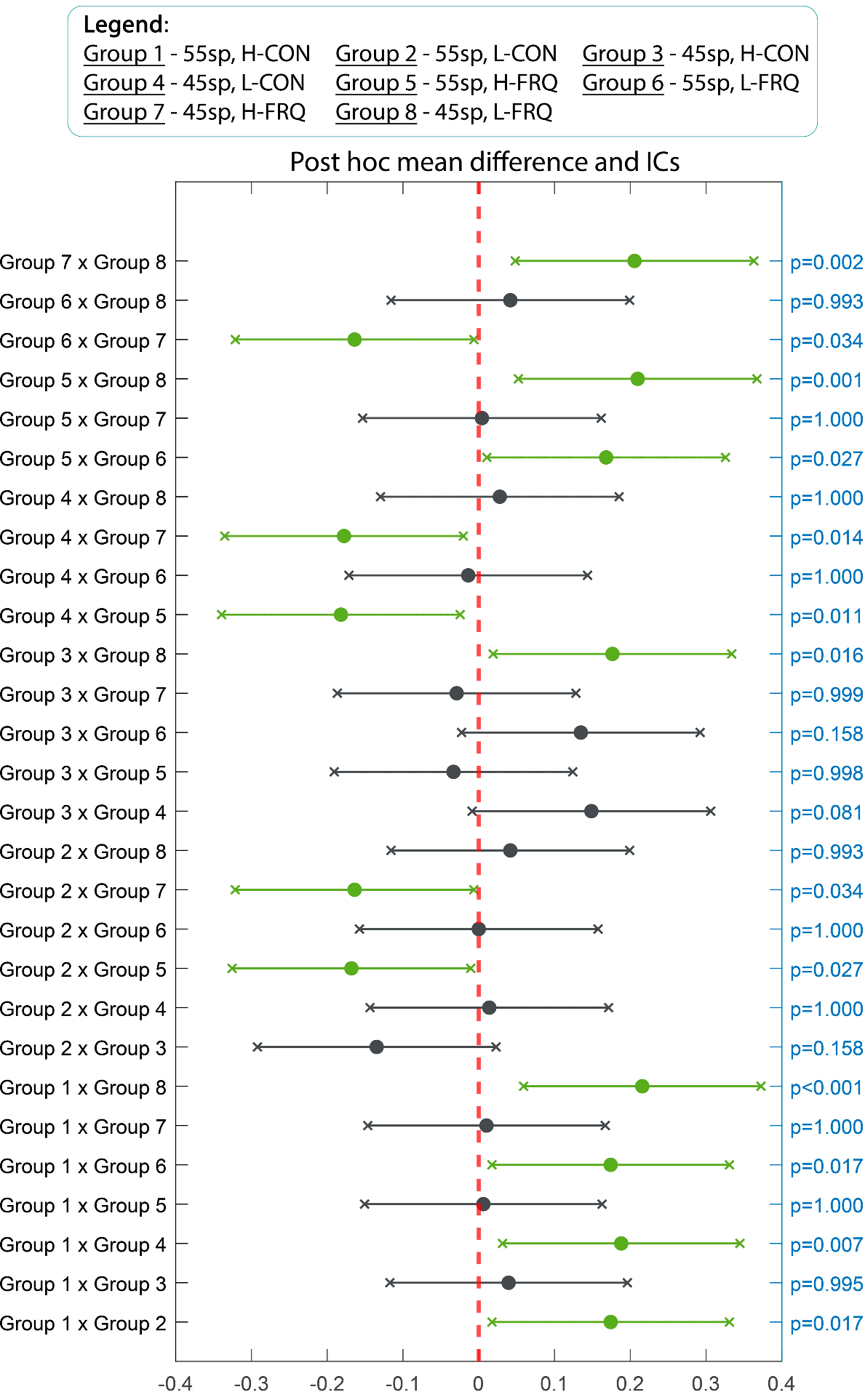
**

**Figure A2. *Post hoc* analysis of the two-way ANOVA (FoV conditions).** Significant differences were found only between groups with different magnitude levels. Figure A1 shows the 95% confidence interval of the mean difference between each multiple comparison, as well as the p-value for each comparison. Groups 1 and 3 present H-FOV conditions while Groups 2 and 4 present L-FOV conditions.


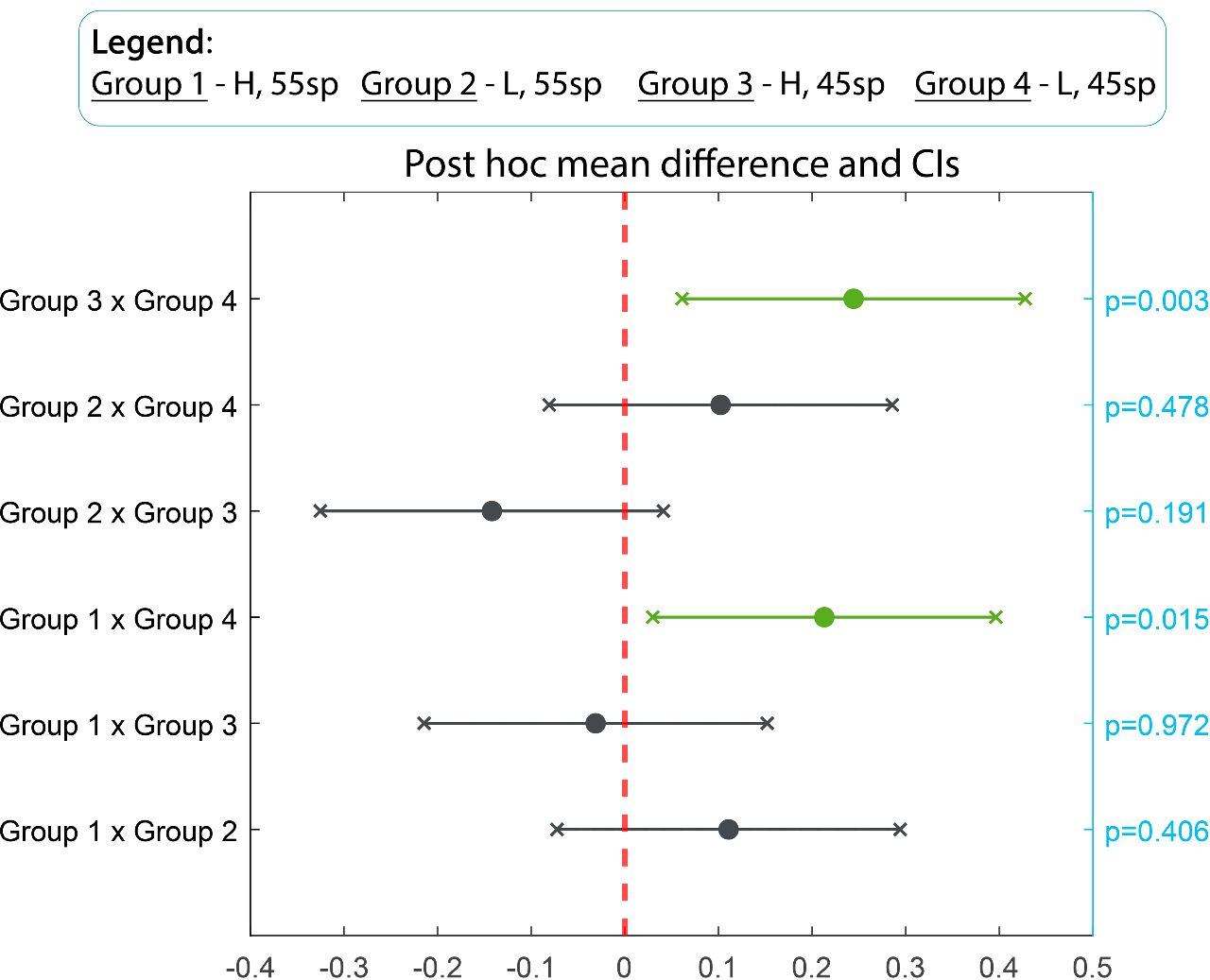


**Follow-up replication of Experiment 1.**

**Statistical Analysis.** A 2$\times$2$\times$2 ANOVA was used to check for significant differences (with *visual feature, magnitude* and *sampling point* as factors). The answer variable was binary (“more than half” or “less than half” of the time elapsed). While the trend observed is the same as in Experiment 1, no significant difference was found for any of the tested factors *(magnitude* F=0.56, p=0.454; *visual feature* F=0.56, p=0.454; *sampling point* F=1.27, p=0.263) probably due to the small sample size (7 participants)*.* Table A3 shows the ANOVA in detail, while Figure A3 shows each post-hoc pairwise comparison between the possible different conditions.

| Source | Sum Sq. | D.F. | Mean Sq. | F | P-value |
| --- | --- | --- | --- | --- | --- |
| Magnitude | 0.143 | 1 | 0.143 | 0.572 | 0.451 |
| Sampling point | 0.321 | 1 | 0.321 | 1.288 | 0.259 |
| Visual Feature | 0.143 | 1 | 0.143 | 0.572 | 0.451 |
| userID | 1.964 | 6 | 0.327 | 1.311 | 0.259 |
| magnitude*sp | 0.571 | 1 | 0.571 | 2.289 | 0.134 |
| magnitude*visFeat | 0.036 | 1 | 0.036 | 0.143 | 0.706 |
| sp*visFeat | 0 | 1 | 0 | 0 | 1 |
| magnitude*sp*visFeat | 0.321 | 1 | 0.321 | 1.288 | 0.259 |
| Error | 24.464 | 98 | 0.250 | - | - |
| Total | 27.964 | 111 | - | - | - |

**Table A3**. 2$\times$2$\times$2 ANOVA of the follow-up replication of Experiment 1 in CDs, with fixed factors: magnitude, sampling point and visual feature. The effect of each participant was considered as a random variable (userID, underlined). First and second order interactions between the fixed variables were considered. Only *magnitude* had a significant effect in the response variable.

**Figure A3. *Post hoc* analysis of the three-way ANOVA (Contrast and Frequency conditions).** No significant differences were found. Figure A3 shows the 95% confidence interval of the mean difference between each multiple comparison, as well as the p-value for each comparison.


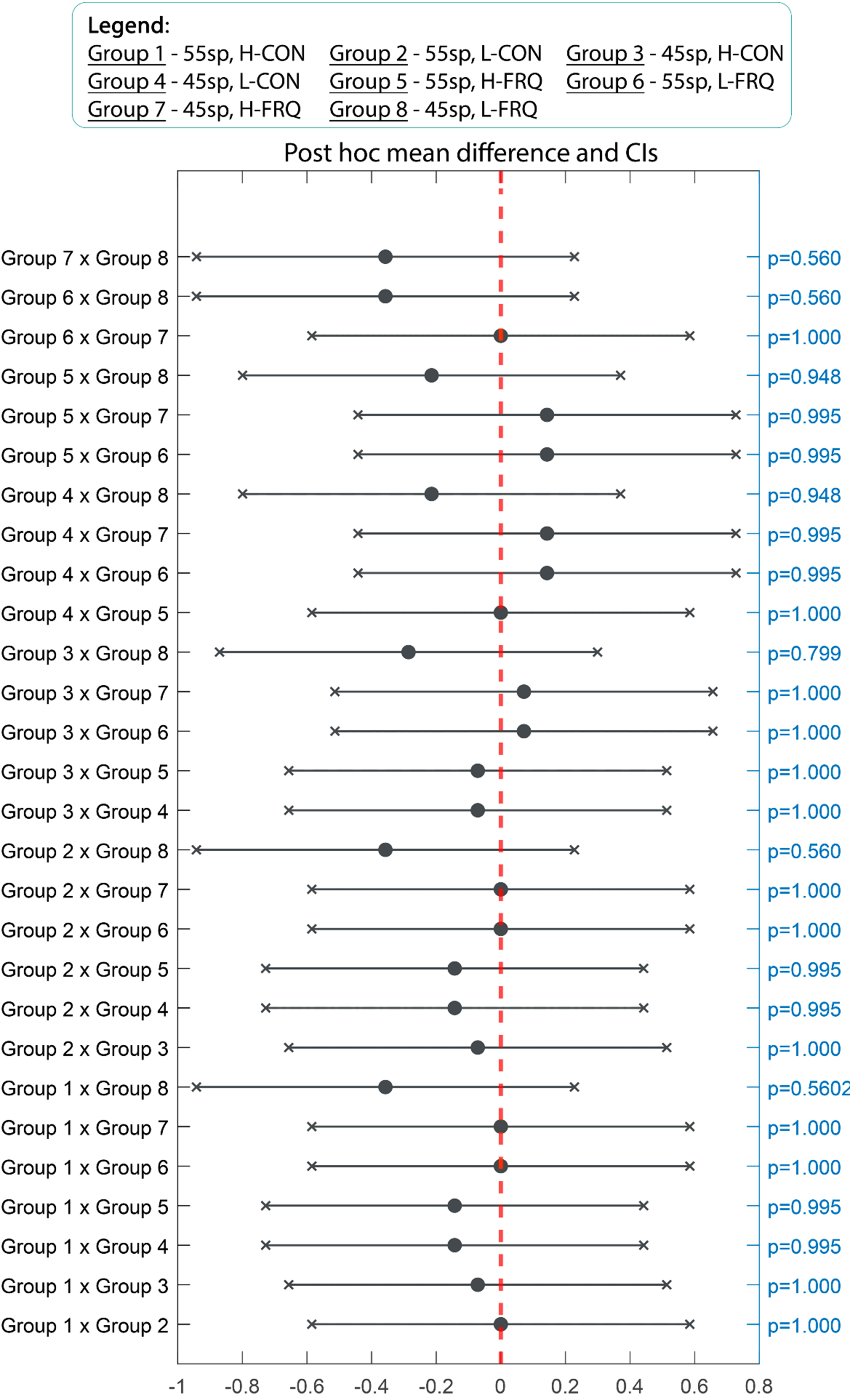


**Experiment 3.**

**Statistical analysis**. An ANOVA was carried out to compare differences in *magnitude* for H-VC and L-VC levels. NASA-TLX questionnaire differences were tested with a t-test. The answer variables were continuous (for the produced durations) or discrete (for the NASA-TLX scores). The independent variable was binary (for low and high magnitude levels of visual complexity). Participants took more time to indicate that 30s had passed in the H-VC condition, suggesting that time was perceived as significantly shorter under higher visual complexity (ratio of 1.38 for H-VC vs 1.10 for L-VC, power=0.407, F=4.98, p=0.0372, partial η2=0.24, normality of distribution checked with Anderson-Darling tests). The complete information about the ANOVA can be found in Table A4. The mean difference between groups H-VC and L-VC was -0.276 (CI [-0.534 -0.018], p=0.0372).

| Source | Sum Sq. | D.F. | Mean Sq. | F | P-value |
| --- | --- | --- | --- | --- | --- |
| Magnitude | 0.420 | 1 | 0.420 | 4.980 | 0.037 |
| Error | 1.685 | 20 | 0.084 | - | - |
| Total | 2.104 | 21 | - | - | - |

**Table A4**. ANOVA of Experiment 3 (high and low visual complexity are the two levels of the single factor -*magnitude*- that is tested). This factor had a significant effect on the answers of the participants.

**Appendix B – GLMM analysis of Experiment 1**

In the following, we report an additional statistical analysis for Experiment 1. In this GLMM we analyze together participant Groups 1.1 and 1.2 (89 participants) to check for significant interactions of the visual features (luminance contrast, temporal frequency and field of view) for completeness. We find consistent results with the previous separated ANOVA analyses: only magnitude has a significant effect on time perception.

The answer variable was binary, and the significance level was established at p=0.05. The information about the GLMM can be found in Table B1. Following standard Matlab nomenclature, this is the model we tested:

answers ~ 1 + (1|userID) + samplingPoint*level + samplingPoint*freq + level*freq + samplingPoint:level:freq

Chi^2-statistic vs. constant model: 81.2, p-value = 2.84e-14

|  | Estimate | SE | T-stat | p-value | CI |
| --- | --- | --- | --- | --- | --- |
| (Intercept) | 0.775 | 0.151 | 5.121 | <0.001 | {0.478, 1.071} |
| Sampling point | -0.175 | 0.201 | -0.868 | 0.385 | {-0.570, 0.220} |
| Magnitude | -0.751 | 0.199 | -3.764 | <0.001 | {-1.142, -0.360} |
| Visual Feature | -0.222 | 0.141 | -1.582 | 0.114 | {-0.498, 0.053} |
| userID | -0.006 | 0.003 | -1.946 | 0.059 | {-0.011, 0.001} |
| magnitude*sp | 0.144 | 0.282 | 0.512 | 0.609 | {-0.408, 0.697} |
| sp*visFeat | 0.152 | 0.182 | 0.837 | 0.403 | {-0.205, 0.510} |
| magnitude*visFeat | 0.121 | 0.183 | 0.660 | 0.509 | {-0.238, 0.480} |
| magnitude*sp*visFeat | -0.344 | 0.261 | -1.320 | 0.187 | {-0.856, 0.167} |

Table B1. GLMM of Experiment 1, considering Groups 1.1 and 1.2, with fixed factors: sampling point, magnitude and visual feature. The effect of each participant was considered as a random variable (userID, underlined).
